# 

**Published:** 1872-01

**Authors:** 


### CONTENTS.

COMMUNICATIONS.

The Extraction of Roots—When is it Indicated? ..... 9
Mechanical Dentistry as an Art—Its Decline ..... 19
Ohio State Dental Society—Address of Dr. Geo. W. Keely, retiring President ..... 23
Prof. Wright and Operative Dentistry ..... 27
Management of a Difficult Case ..... 30

PROCEEDINGS OF SOCIETIES.

Annual Meeting of the Ohio State Dental Society ..... 31
The Dentists—Kentucky State Dental Association—How it was Organized, by whom, and for what purpose ..... 42

SELECTIONS.

Valedictory Address delivered to the Graduating Class of Belleview Medical College, March 2, 1871 ..... 45
Poisoned by Mercury from a Tooth Filling ..... 47
Safe Anæsthesia ..... 48
Anæsthetics ..... 49
Annual Meeting ..... 50

EDITORIAL.

Dental Education ..... 51

